# OVERCOMING TENSIONS IN CULTURALLY RESPONSIVE RESEARCH WITH LGBTQ+ OLDER ADULTS OF COLOR

**DOI:** 10.1093/geroni/igad104.0257

**Published:** 2023-12-21

**Authors:** Austin Oswald, Lujira Cooper, Aundaray Guess

**Affiliations:** University of Washington, Seattle, Washington, United States; Services and Advocacy for GLBT Elders, New York City, New York, United States; GRIOT Circle, Brooklyn, New York, United States

## Abstract

Lesbian, gay, bisexual, transgender, and queer plus (LGBTQ+) older adults of color are underrepresented in gerontological scholarship due to structural factors that limit their research participation. This presentation applies critical reflexivity to weave together the narratives of a gerontological researcher, service provider, and older adult who co-constructed a research project committed to improving the visibility of LGBTQ+ older adults of color in gerontology scholarship. Through the lens of queer failure, we analyze personal narratives documenting our process designing and implementing a participatory action research project to uncover pivotal moments that shifted the course of the research in unexpected, yet critically important ways. Three themes emerged that illuminate key epistemic tensions associated with culturally responsive research with LGBTQ+ older adults of color, specifically navigating cross cultural tensions, intergenerational tensions, and pandemic related tensions. These findings highlight the limitations of traditional research methods which fail to account for historic and contemporary experiences that may shape perceptions of researchers and individuals’ willingness to participate in research. Working through these tensions in solidarity contributed to the revision and improvement of traditional methods by developing culturally responsive approaches to research with LGBTQ+ older adults of color. Embodying a participatory, reflexive, and collaborative approach to inquiry has the potential to challenge and transform hegemonic scientific discourse that marginalizes certain communities. This presentation ends by offering implications for research that may support the development of culturally responsive methods for underrepresented communities in which future studies can expand upon, so long as the community is in the lead.

